# Screening, optimization and artificial recombination of dsRNA fragments for RNAi-mediated pest resistance in *Apolygus lucorum*

**DOI:** 10.3389/finsc.2026.1912427

**Published:** 2026-08-18

**Authors:** Juan Luo, Qianqian Guo, Ruyi Hu, Mengjun Zhang, Guirong Wang, Bin Yang

**Affiliations:** 1 State Key Laboratory for Biology of Plant Diseases and Insect Pests, Institute of Plant Protection, Chinese Academy of Agricultural Sciences, Beijing, China; 2 Anhui Province Key Laboratory of Crop Integrated Pest Management/Key Laboratory of Biology and Sustainable Management of Plant Diseases and Pests of Anhui Higher Education Institutes, College of Plant Protection, Anhui Agricultural University, Hefei, China; 3 Guangdong Laboratory for Lingnan Modern Agriculture, Genome Analysis Laboratory of the Ministry of Agriculture, Agricultural Genomics Institute at Shenzhen, Chinese Academy of Agricultural Sciences, Shenzhen, China

**Keywords:** *Apolygus lucorum*, artificially recombinant dsRNA, dsRNA optimization, off-target effect, RNAi-based insect resistance technology

## Abstract

RNA interference (RNAi) is an eco-friendly strategy for pest management, with double-stranded RNA (dsRNA) as the core functional component. In this study, three RNAi target genes (*Ubx*, *wupA* and *Dpp*) with strong lethal effects on *Apolygus lucorum* were screened via microinjection. The 7-day cumulative mortalities were 56.67 ± 3.33% for ds*Ubx*, 94.44 ± 1.11% for ds*wupA* and 92.22 ± 1.11% for ds*Dpp*. We optimized dsRNA sequences by removing conserved sequences in non-target organisms based on homology alignment and off-target risk analysis. The optimized fragments *dswupA-OTE* and *dsDpp-OTE* still exhibited high insecticidal activity, with 7-day cumulative mortalities of 77.78 ± 2.94% and 70.00 ± 1.93%, respectively. We also evaluated the effects of dsRNA length and target sites on RNAi efficiency and screened potent short dsRNA fragments. Novel artificially recombinant dsRNAs were constructed by assembling effective short fragments from different genes, which retained strong insecticidal activity despite shorter sequence length. This study verifies the feasibility of multi-target recombinant dsRNA for pest control and provides a theoretical basis for developing multi-gene RNAi technologies against *A. lucorum*.

## 1 Introduction

RNA interference (RNAi) is a conserved post-transcriptional gene silencing mechanism triggered by double-stranded RNA (dsRNA), and has emerged as a promising tool for sustainable pest management. (1). Its agricultural feasibility has been demonstrated by several commercial products, such as the transgenic maize MON87411 in 2017 and the sprayable dsRNA biopesticide Ledprona and Vadescana in 2023 and 2025, respectively (2–4). In 2026, the Institute for the Control of Agrochemicals (ICAMA) under China’s Ministry of Agriculture and Rural Affairs issued a public notice indicating its intention to approve the country’s first RNA pesticide. These successful cases lay a solid foundation for the commercialization of RNAi-based pest control technologies. However, the successful application of RNAi still relies on continuous discovery of efficient target genes and optimization of dsRNA delivery parameters, especially for pests that are not adequately controlled by conventional approaches.

dsRNA length and target sequence position are two key parameters that profoundly influence RNAi efficiency, and their effects have been extensively documented across different insect species, highlighting the need for systematic optimization for each target pest. dsRNA length markedly affects RNAi efficiency, with long dsRNAs generally exhibiting stronger silencing than siRNAs (5), though optimal length varies among species (6). In *Drosophila*, 400–540 bp dsRNAs effectively silenced *cyclin E*, whereas 200–300 bp fragments were weak and 50–100 bp inactive (7), and the minimum efficient length in S2 cells was 211 bp (8). In *Manduca sexta*, a 2222 bp dsRNA gave higher mortality (56.8%) than a 259 bp one (47.8%) (9). However, short dsRNAs (<40 nt) can also be effective via oral delivery (10), 21 bp injection silenced a salivary gene in aphids (11), and 15–25 bp siRNAs silenced two termite genes (12). Thus, dsRNA length should be optimized per gene considering efficiency and off-target risks. Target sequence position also modulates RNAi performance: fragments covering the 3′ region of *V-ATPase E* were more active in *Nilaparvata lugens* (13), whereas in *A. lucorum* different regions of *JHEH* showed comparable effects (14) indicating species- and gene-dependent influences of target position.

In addition to optimizing dsRNA design parameters, multi-target combination strategies have been developed to overcome the limited efficacy and biosafety concerns of single-gene dsRNAs. By fusing short effective fragments from multiple essential genes, this approach simultaneously disrupts several key pathways, achieving high mortality at low doses with reduced off-target effects (15). For example, fusion dsRNAs targeting 3–6 salivary protein genes in aphids caused 60% mortality in *Myzus persicae*, markedly higher than the 28–38% from single-gene dsRNAs (15). In *M. persicae* and *Bemisia tabaci*, two fusion constructs (*dsAbd-A-Rpl27a-H3* and *dsScr-Abd-A-H3*) achieved peak mortalities of 59.08% and 45.33%, respectively, with low off-target risk to the predator *Propylaea japonica* (16). In *A. lucorum*, a dual-target dsRNA against *ECR-A* and *Tre-1* delivered with SPc nanocarriers gave 83% nymph mortality upon spray application, significantly exceeding single-target treatments (17). These findings support the multi-target strategy as a promising direction for RNAi-based pest control.

The mirid bug *Apolygus lucorum* (Hemiptera: Miridae) is a polyphagous pest that infests over 200 host plants, including cotton, fruit trees and vegetables (18, 19). In cotton fields, the widespread cultivation of *Bacillus thuringiensis* (Bt) transgenic crops has effectively suppressed lepidopteran pests but provides poor control against hemipterans due to the lack of functional Bt receptors (20, 21). Consequently, *A. lucorum* has become a dominant pest, cause abscission or malformation of leaves, flowers and fruits (22). The management for *A. lucorum* still relies heavily on chemical insecticides, which have led to resistance development and environmental concerns (23, 24). Therefore, novel eco-friendly strategies are urgently needed. Previous studies have explored RNAi‐based control of *A. lucorum* by targeting genes involved in metabolism or stress responses, and have achieved moderate lethal effects (25–27). For example, microinjection-based RNAi screens in *A. lucorum* have identified multiple lethal targets (e.g., *Alucβ-actin*, V-ATPase subunits, *AlLIM*, and *Al6*), with *Alucβ-actin* giving the highest mortality (82.32%), *AlLIM* yielding 38–81% mortality, and silencing *Al6* significantly reducing feeding, weight, and survival. However, several critical gaps remain, specifically that the influence of dsRNA design parameters (fragment length and target sequence position) on RNAi efficiency has not been optimized for *A. lucorum* despite their known species-dependent variability, and that single-target dsRNAs often confer limited mortality whereas multi-target combination strategies that simultaneously silence multiple essential genes may offer improved efficacy.

To address these gaps, we selected four key genes that are integral to insect development and signaling pathways: *Ubx* (Ultrabithorax), which represses wing-related genes and activates haltere-specific genes (28–31); *wupA*, encoding troponin critical for muscle development (32, 33); *Dpp* (Decapentaplegic), involved in embryonic patterning, appendage differentiation and oogenesis (34–38); and *PP1*, a serine/threonine phosphatase with diverse regulatory functions (39). These genes have not been previously examined as RNAi targets in *A. lucorum*, and their essential roles suggest that their knockdown could cause strong deleterious effects. In addition, we designed dsRNAs of varying lengths and targeting different regions of these genes to determine the optimal design for maximal silencing. We also constructed a fused dsRNA combining effective fragments from multiple targets to test whether a multi-target approach could produce synergistic mortality.

## 2 Materials and methods

### 2.1 Test insects

*A. lucorum* were collected from the experimental field of the Institute of Plant Protection, Chinese Academy of Agricultural Sciences in Langfang, Hebei Province. The insects were reared in plastic containers (20 × 10 × 6 cm³) with corn and kidney beans as food. Rearing conditions were set at 27 ± 1 °C, relative humidity of 60 ± 5%, and a photoperiod of 15 h light: 9 h dark (L:D = 15:9). Healthy third-instar nymphs were used in all experiments.

### 2.2 Screening and sequence analysis of target genes

Four key genes associated with insect development, namely *PP1*, *Ubx*, *wupA* and *Dpp*, were selected in this study. Their nucleotide sequences were retrieved from the NCBI database with the following accession numbers: *PP1* (XM_014431150.1), *Ubx* (XM_024359729.1), *Dpp* (NM_164488.2) and *wupA* (MH001571.1). All sequences were aligned against the transcriptome data of *A. lucorum* established in our laboratory. The *GFP* gene (GenBank Accession No.U76561) was used as the negative control.

### 2.3 Prediction of off-target sequences

Referring to the criteria described by Kulkarni et al. (40), a continuous 19 bp homologous region between dsRNA and mRNA of non-target organisms was defined as an off-target site. Accordingly, sequences containing continuous 19 bp identical fragments with genes from non-target organisms were regarded as potential off-target sequences in this study.

### 2.4 Total RNA extraction and cDNA synthesis

Whole adults of *A. lucorum* (3–5 individuals) were collected, and total RNA was extracted using Trizol reagent (Invitrogen, USA) following the manufacturer’s protocols. RNA integrity was assessed via 1% agarose gel electrophoresis, while concentration and purity were determined using a NanoDrop 2000 spectrophotometer. First-strand cDNA was synthesized from 1 μg of total RNA with the RevertAid First Strand cDNA Synthesis Kit (Fermentas, USA). The obtained cDNA was stored at −20 °C for subsequent use.

### 2.5 Preparation and sequence optimization of dsRNA templates

Specific primers were designed using Premier 5.0 software based on the sequences of *PP1*, *Ubx*, *wupA* and *Dpp* from *A. lucorum*. Target fragments were amplified by PCR using the synthesized cDNA as the template. The PCR products were purified by gel extraction, ligated into the pEASY-Blunt vector (TransGen Biotech, Beijing), and transformed into *Escherichia coli* Trans1-T1 competent cells. Positive clones were selected and verified by sequencing. Referring to the criteria established for *Drosophila melanogaster* by Kulkarni et al. (40), continuous identical sequences of 19 bp or longer between dsRNA and genes of non-target organisms were defined as potential off-target sites. The original dsRNA target sequences of *Ubx*, *wupA* and *Dpp* were subjected to blastn searches in the NCBI database. Homology with other organisms, especially beneficial non-target species, was manually analyzed and recorded. Regions containing continuous identical sequences longer than 19 bp shared with non-pest organisms were identified as risky sites and removed. By contrast, regions homologous only to other pest species, designated as universal target site, were retained. After optimization, new target fragments including *wupA*-OTE, *Dpp*-OTE and a series of *Ubx*-ss fragments were obtained.

### 2.6 Preparation of templates with T7 promoter and dsRNA synthesis

PCR amplification was performed using sequence-verified plasmids as templates and specific primers containing the T7 promoter sequence (TAATACGACTCACTATAGGGAGA). The total volume of each PCR reaction was 200 μL with 2× Easy Taq Mix applied. Amplified products were purified via phenol-chloroform extraction, air-dried and resuspended in RNase-free water to serve as templates for *in vitro* dsRNA synthesis. dsRNA was synthesized *in vitro* following the protocols of the MEGAscript T7 Transcription Kit (Ambion, USA). An 800 μL reaction system containing template DNA, NTP mix, reaction buffer and T7 enzyme mixture was incubated in a metal bath at 37 °C for 4 h or overnight. Subsequently, the products were purified by phenol-chloroform extraction and ethanol precipitation. After drying, the pellets were dissolved in an appropriate volume of RNase-free water. The integrity and concentration of dsRNA were examined by 1% agarose gel electrophoresis and NanoDrop spectrophotometry. Qualified dsRNA samples were stored at −80 °C for subsequent experiments.

### 2.7 Microinjection of dsRNA and phenotype observation

The synthesized dsRNA was diluted to working concentrations with RNase-free water. A concentration of 5 μg/μL was used for routine screening, while four concentration gradients (0.5, 1, 3 and 5 μg/μL) were set for the artificially recombinant sequence *Udw*. Uniform third-instar nymphs were lightly anesthetized with CO_2_ and placed ventral side up on a 1% agarose gel plate. A total volume of 41.4 nL dsRNA solution was injected into the intersegmental membrane between the first abdominal segment and thorax using a Nanoliter 2010 microinjector (World Precision Instruments, USA). Each treatment contained 30 nymphs with three biological replicates. Nymphs injected with equal concentrations of ds*GFP* served as the control group. After injection, the insects were transferred to Petri dishes lined with moist filter paper, fed with fresh corn kernels and reared under the original laboratory conditions. Nymph mortality was recorded daily for seven consecutive days, and the cumulative mortality as well as relative mortality were calculated accordingly.

### 2.8 Strategy for construction of recombinant Udw dsRNA

Through preliminary screening of highly lethal small fragments targeting the three genes *Ubx*, *Dpp* and *wupA*, four potent lethal small fragments were obtained, namely *Ubx*-ss-1, *Dpp*-ss-2, *wupA*-ss-1 and *wupA*-ss-2. Since *wupA*-ss-1 and *wupA*-ss-2 are adjacent fragments with overlapping sequences, they were collectively designated *wupA*-ss-12 in this study. Subsequently, the three fragments were concatenated in the order of *Ubx*-ss-1 (511–585 bp, 75 bp in length), *Dpp*-ss-2 (730–794 bp, 65 bp in length) and *wupA*-ss-12 (206–327 bp, 122 bp in length). The resulting fused sequence was named *Udw*, with a total length of 262 bp. The junction sites of this construct were annotated against the NCBI reference sequence.

The target fragment was synthesized by Sangon Biotech. The synthetic fragment was cloned into the pEASY-Blunt vector (TransGen Biotech, Beijing, China), followed by transformation into *Trans1-T1* competent cells. Positive clones were screened and verified via Sanger sequencing. Sequencing-confirmed plasmids were used as templates for PCR amplification with specific primers appended with the T7 promoter sequence (TAATACGACTCACTATAGGGAGA). The 200 μL PCR system was prepared using 2× Easy Taq Mix. Amplified products were purified by phenol-chloroform extraction, vacuum-dried, and dissolved in RNase-free water to serve as templates for *in vitro* dsRNA synthesis. *In vitro* transcription of dsRNA was performed following the manufacturer’s instructions of the MEGAscript T7 Transcription Kit (Ambion, USA). An 800 μL reaction system containing template DNA, NTP mix, reaction buffer and T7 enzyme mix was incubated at 37 °C in a metal bath for 4 h or overnight. After transcription, the dsRNA products were purified via phenol-chloroform extraction and ethanol precipitation, air-dried, and resuspended in an appropriate volume of RNase-free water. The integrity and concentration of dsRNA were examined by 1% agarose gel electrophoresis and NanoDrop spectrophotometry, and qualified dsRNA aliquots were stored at −80 °C for subsequent use.

### 2.9 Data statistics and analysis

Raw experimental data were organized using Microsoft Excel and statistically analyzed with GraphPad Prism. Student’s t-test was applied to assess significant differences (*P < 0.05, **P < 0.01, ***P < 0.001, ****P < 0.0001; ns indicates no significant difference). All data in figures are presented as Mean ± standard error of the mean (SEM).

## 3 Results and analysis

### 3.1 Screening of target genes

**Figure 1:**
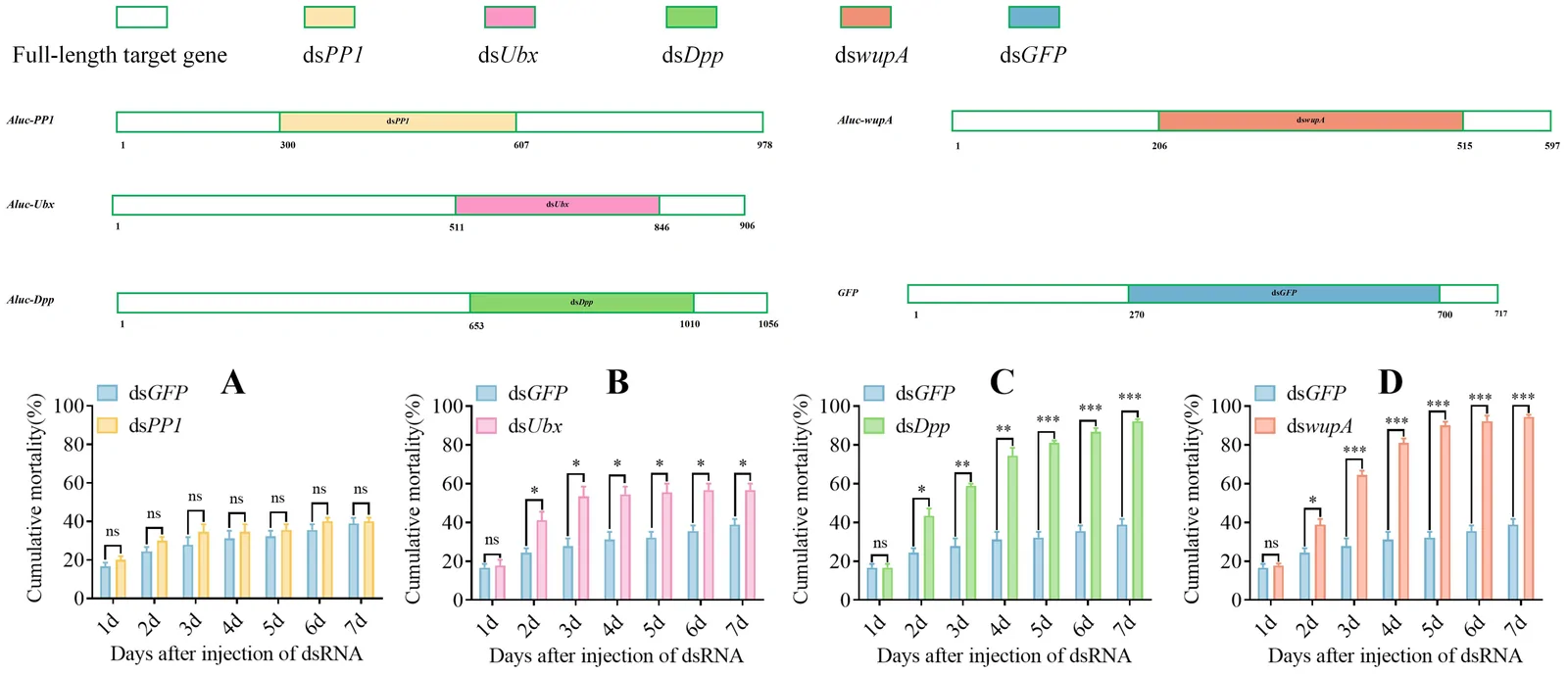
Screening of RNAi target genes for pest control in *A. lucorum*. Cumulative mortality of 3rd-instar *A. lucorum* nymphs at 7 days post microinjection of dsRNA. dsRNA was injected at a concentration of 5 μg/μL with an injection volume of 41.1 nL. **(A)** ds*PP1*; **(B)** ds*Ubx*; **(C)** ds*wupA*; **(D)** ds*Dpp* (*p < 0.05, **p < 0.01, ***p < 0.001, and ns was no significant difference).

To screen effective RNAi target genes for the control of *A. lucorum*, dsRNA fragments targeting *PP1* (308 bp), *Ubx* (336 bp), *wupA* (310 bp) and *Dpp* (358 bp) were separately delivered into third-instar nymphs via microinjection, with ds*GFP* as the control. Cumulative mortality was recorded daily for seven consecutive days. The results are shown in **Figure 1**. The 7-day cumulative mortality of nymphs injected with ds*Ubx* was 56.67 ± 3.33%, which was significantly higher than that of the control group (*P* < 0.05). Treatments with ds*wupA* and ds*Dpp* produced strong lethal effects, with cumulative mortalities reaching 94.44 ± 1.11% and 92.22 ± 1.11%, respectively, both showing extremely significant differences compared with the control (*P* < 0.001). By contrast, the 7-day cumulative mortality in the ds*PP1* treatment group was only 40.00 ± 1.92%, with no significant difference from the control.

### 3.2 Off-target analysis of RNAi target fragments in *A. lucorum*

Homology searches were performed on the dsRNA regions of *Ubx*, *wupA* and *Dpp* via the NCBI database. Sequences containing continuous identical fragments longer than 19 bp shared with non-target organisms were defined and recorded as off-target sites. Sites homologous exclusively to pest species were designated as universal target site and marked in green in the figure. By contrast, sites that could cause off-target effects on non-pest organisms were defined as risky sites and marked in red, with the names of corresponding non-pest species labeled in red font. As shown in **Supplementary Appendix 1**. The detailed information of the species is shown in **Supplementary Table 1**, three risky sites were identified in the *Ubx* fragment, located at 586–614 bp, 656–692 bp and 730–838 bp. Sequences at these sites shared high homology with diverse non-target organisms, including ants such as *Pseudomyrmex gracilis* and *Wasmannia auropunctata*, fish including *Labrus bergylta*, bees such as *Bombus terrestris* and *Osmia bicornis*. One universal target site (345–436 bp) and one risky site (447–494 bp) were detected in *wupA*. The universal target site shared homology with *Thrips palmi*. The risky site was homologous to multiple insect pests, including *Plutella xylostella*, *Amyelois transitella*, *Manduca sexta*, *Loxostege sticticalis*, *Dendroctonus ponderosae*, *Thrips palmi* and *Frankliniella occidentalis*. For the *Dpp* fragment, two universal target site were found at 755–806 bp and 820–848 bp, which were homologous to aphid species including *Sipha flava*, *Melanaphis sacchari* and *Diuraphis noxia*, as well as *Cimex lectularius*. One risky site (935–953 bp) was also identified, with homologous sequences present in fish such as *Centropristis striata* and *Cololabis saira*, and birds including *Malurus melanocephalus*, *Prinia subflava* and *Ficedula albicollis*.

### 3.3 Insecticidal activity verification of the longest fragments of candidate genes after removing risky sites

**Figure 2:**
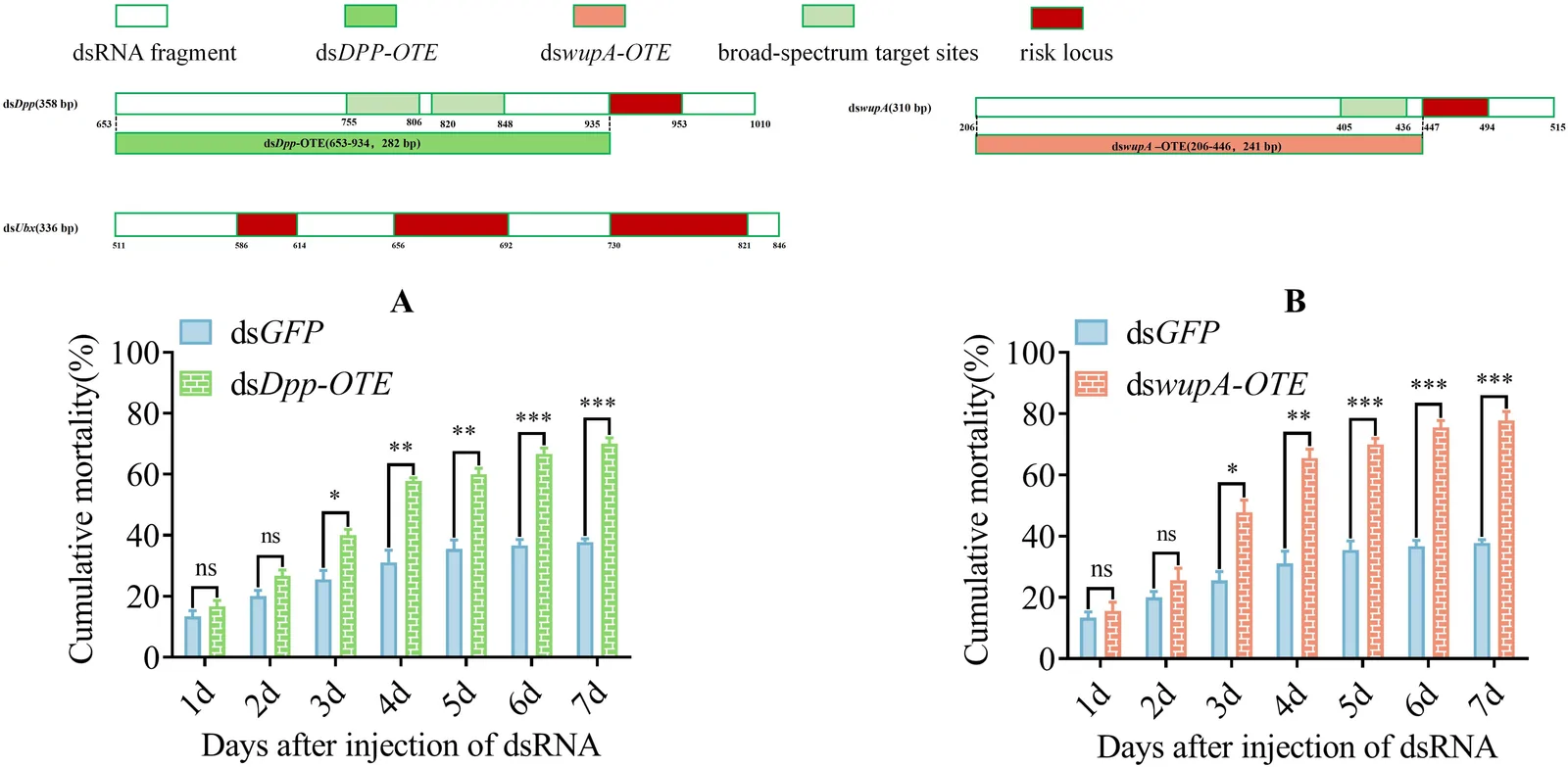
Verification of efficacy after off-target optimization of *wupA* and *Dpp.* Cumulative mortality of 3rd-instar *A. lucorum* nymphs at 7 days post microinjection of optimized dsRNA. The injection concentration of dsRNA was 5 μg/μL, with an injection volume of 41.1 nL. **(A)** ds*Dpp*-OTE; **(B)** ds*wupA*-OTE. (*p < 0.05, **p < 0.01, ***p < 0.001, and ns was no significant difference).

*Ubx*, *wupA* and *Dpp* were selected for subsequent experiments. After the risky sites were deleted, the longest remaining fragments of *Ubx*, *wupA* and *Dpp* were 75 bp (511–585 bp), 241 bp (206–446 bp) and 282 bp (653–934 bp), respectively. Since *wupA*-OTE (241 bp) and *Dpp*-OTE (282 bp) were longer than 200 bp, their RNAi effects were verified firstly. As shown in **Figure 2**, the 7-day cumulative mortality of *Apolygus lucorum* reached 77.78 ± 2.94% (*P* < 0.001) and 70.00 ± 1.93% (*P* < 0.001) after injection with ds*wupA*-OTE and ds*Dpp*-OTE, respectively. Both values were extremely significantly higher than that of the control group, indicating that the optimized fragments retained strong insecticidal activity. After off-target analysis of *Ubx*, only three short sequences remained following the removal of risky sites, with lengths of 75 bp, 41 bp and 37 bp. All of them were shorter than 100 bp, so no further verification was performed for long fragments of *Ubx*.

### 3.4 Effects of dsRNA length and target position on RNAi efficiency

**Figure 3:**
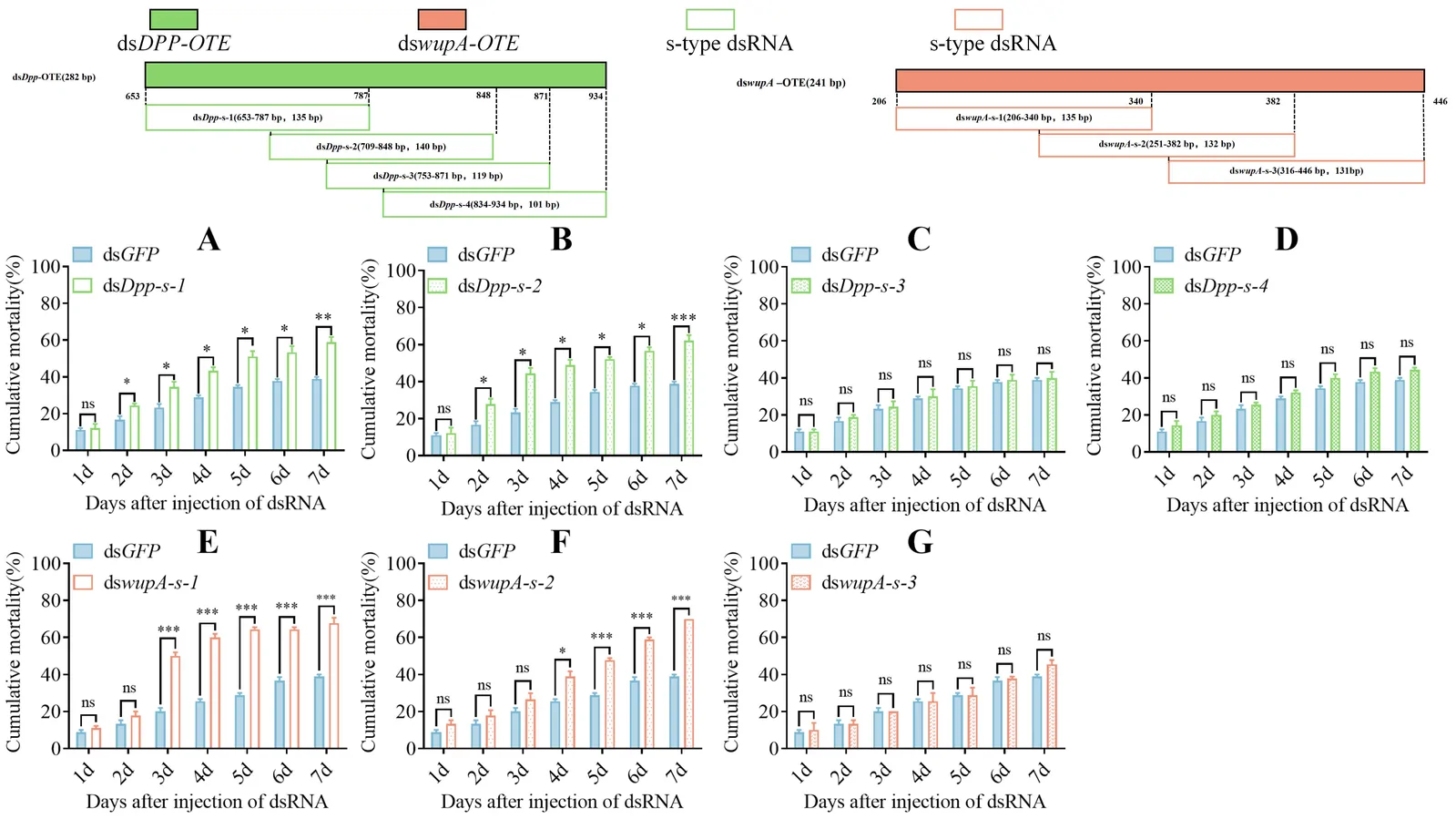
Screening of effective lethal s-type short fragments. Cumulative mortality of 3rd-instar *A. lucorum* nymphs at 7 days post microinjection of short-length (s-class) dsRNA. dsRNA was injected at 5 μg/μL with an injection volume of 41.1 nL. **(A)** ds*Dpp*-s-1; **(B)** ds*Dpp*-s-2; **(C)** ds*Dpp*-s-3; **(D)** ds*Dpp*-s-4; **(E)** ds*wupA*-s-1; **(F)** ds*wupA*-s-2; **(G)** ds*wupA*-s-3 (*p < 0.05, **p < 0.01, ***p < 0.001, and ns was no significant difference).

To explore the effects of dsRNA length and target position on RNAi efficiency and screen effective short dsRNA fragments, *Dpp*-OTE (282 bp) and *wupA*-OTE (241 bp) were further divided into two types of fragments: s-type fragments (100–150 bp) and ss-type fragments (30–90 bp). As shown in **Figure 3**, for the *Dpp* gene, two s-type fragments ds*Dpp*-s-1 (135 bp) and ds*Dpp*-s-2 (140 bp) caused significant mortality in *Apolygus lucorum*. Their 7-day cumulative mortalities were 58.89 ± 2.94% (*P* < 0.01) and 62.22 ± 2.94% (*P* < 0.001), respectively. By contrast, the 7-day cumulative mortalities of the groups treated with ds*Dpp*-s-3 and ds*Dpp*-s-4 were 38.89 ± 2.94% and 44.44 ± 1.11%, showing no significant difference compared with the ds*GFP* control group (38.89 ± 1.11%). For the *wupA* gene, the 7-day cumulative mortalities of nymphs injected with ds*wupA*-s-1 (135 bp) and ds*wupA*-s-2 (132 bp) reached 67.78 ± 2.94% (*P* < 0.0001) and 70.00 ± 0.00% (*P* < 0.001), respectively. The mortality of the ds*wupA*-s-3 treatment group was 45.55 ± 2.22%, which was not significantly different from the control group (38.89 ± 1.11%).

**Figure 4:**
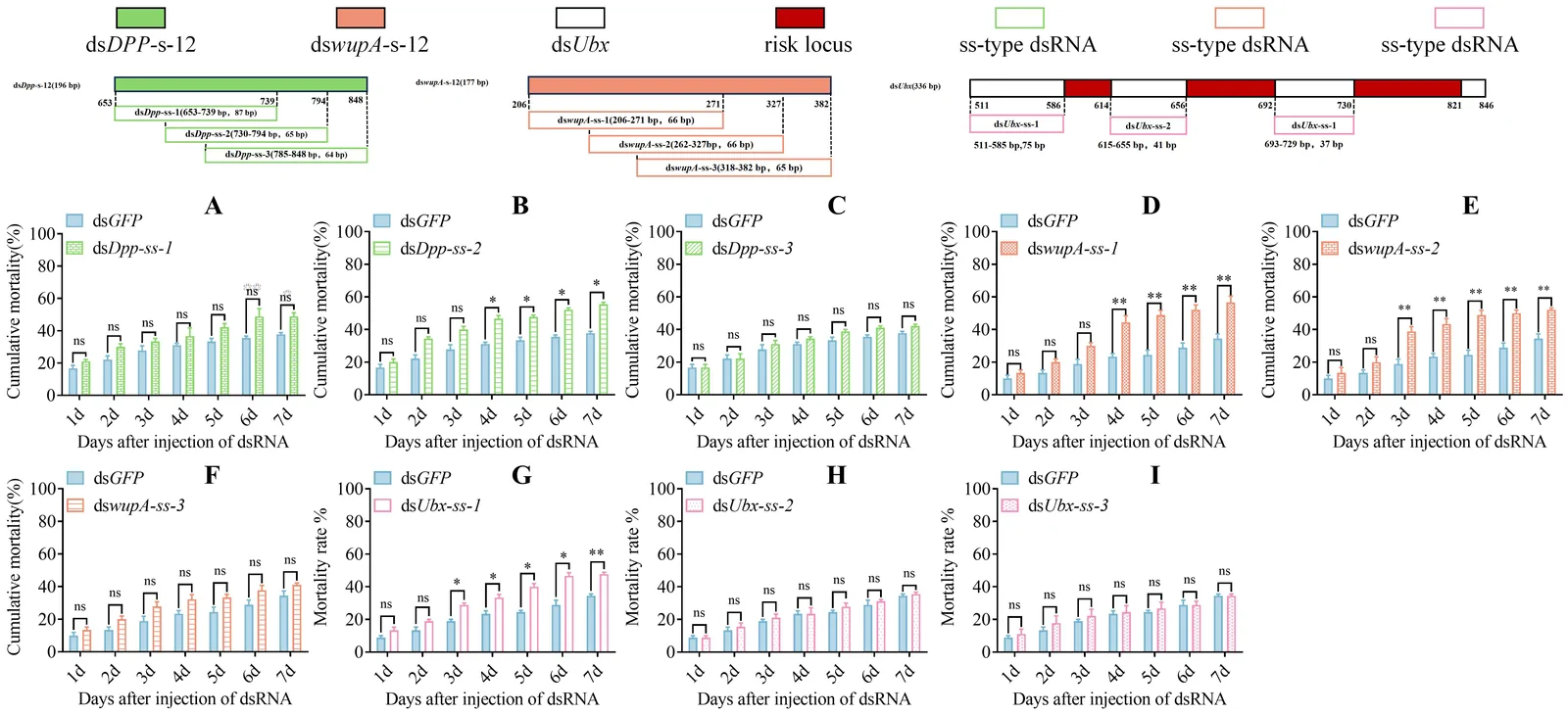
Screening of effective lethal ss-type short fragments. Cumulative mortality of 3rd-instar *A. lucorum* nymphs at 7 days post microinjection of ss-class dsRNA. dsRNA was injected at a concentration of 5 μg/μL with an injection volume of 41.1 nL. **(A)** ds*Dpp*-ss-1; **(B)** ds*Dpp*-ss-2; **(C)** ds*Dpp*-ss-3; **(D)** ds*wupA*-ss-1; **(E)** ds*wupA*-ss-2; **(F)** ds*wupA*-ss-3; **(G)** ds*Ubx*-ss-1; **(H)** ds*Ubx*-ss-2; **(I)** ds*Ubx*-ss-3 (*p < 0.05, **p < 0.01, and ns was no significant difference).

As shown in **Figure 4**, the effective regions covered by *Dpp*-s-1 and *Dpp*-s-2 were further divided into ss-type fragments. Only ds*Dpp*-ss-2 (65 bp) exhibited significant insecticidal activity, with a 7-day cumulative mortality of 55.56 ± 2.94% (*P* < 0.05). The 7-day cumulative mortalities of ds*Dpp*-ss-1 and ds*Dpp*-ss-3 were 48.89 ± 4.01% and 42.22 ± 2.94%, respectively, both showing no significant difference compared with the ds*GFP* control group (36.67 ± 1.93%). After the effective region of the *wupA* gene was truncated into ss-type fragments, both ds*wupA*-ss-1 (66 bp) and ds*wupA*-ss-2 (66 bp) retained prominent insecticidal effects. Their 7-day cumulative mortalities were 56.67 ± 3.85% (*P* < 0.01) and 52.22 ± 1.11% (*P* < 0.01). The mortality of the ds*wupA*-ss-3 treatment group was 41.11 ± 1.11%, which was not significantly different from the control group (34.44 ± 2.94%). Three dsRNA fragments were designed based on the remaining sequences of *Ubx*, namely *Ubx*-ss-1 (511–585 bp, 75 bp), *Ubx*-ss-2 (615–655 bp, 41 bp) and *Ubx*-ss-3 (693–729 bp, 37 bp). The highest 7-day mortality of 47.78 ± 1.11% (*P* < 0.01) was observed in the ds*Ubx*-ss-1 group. The 7-day cumulative mortalities of the ds*Ubx*-ss-2 and ds*Ubx*-ss-3 groups were 35.56 ± 1.11% and 34.44 ± 1.11%, respectively, with no significant difference relative to the ds*GFP* control (34.44 ± 1.11%).

### 3.5 Construction and insecticidal efficacy evaluation of artificial recombinant multi-target dsRNA (*Udw*)

Based on the screening results of effective short dsRNA fragments, *Ubx*-ss-1 (75 bp), *Dpp*-ss-2 (65 bp) and *wupA*-ss-12 (122 bp, assembled by connecting *wupA*-ss-1 and *wupA*-ss-2) were artificially concatenated to generate a novel recombinant dsRNA of 262 bp, designated as Udw. BLAST analysis confirmed no additional off-target sites in this recombinant sequence.

**Figure 5:**
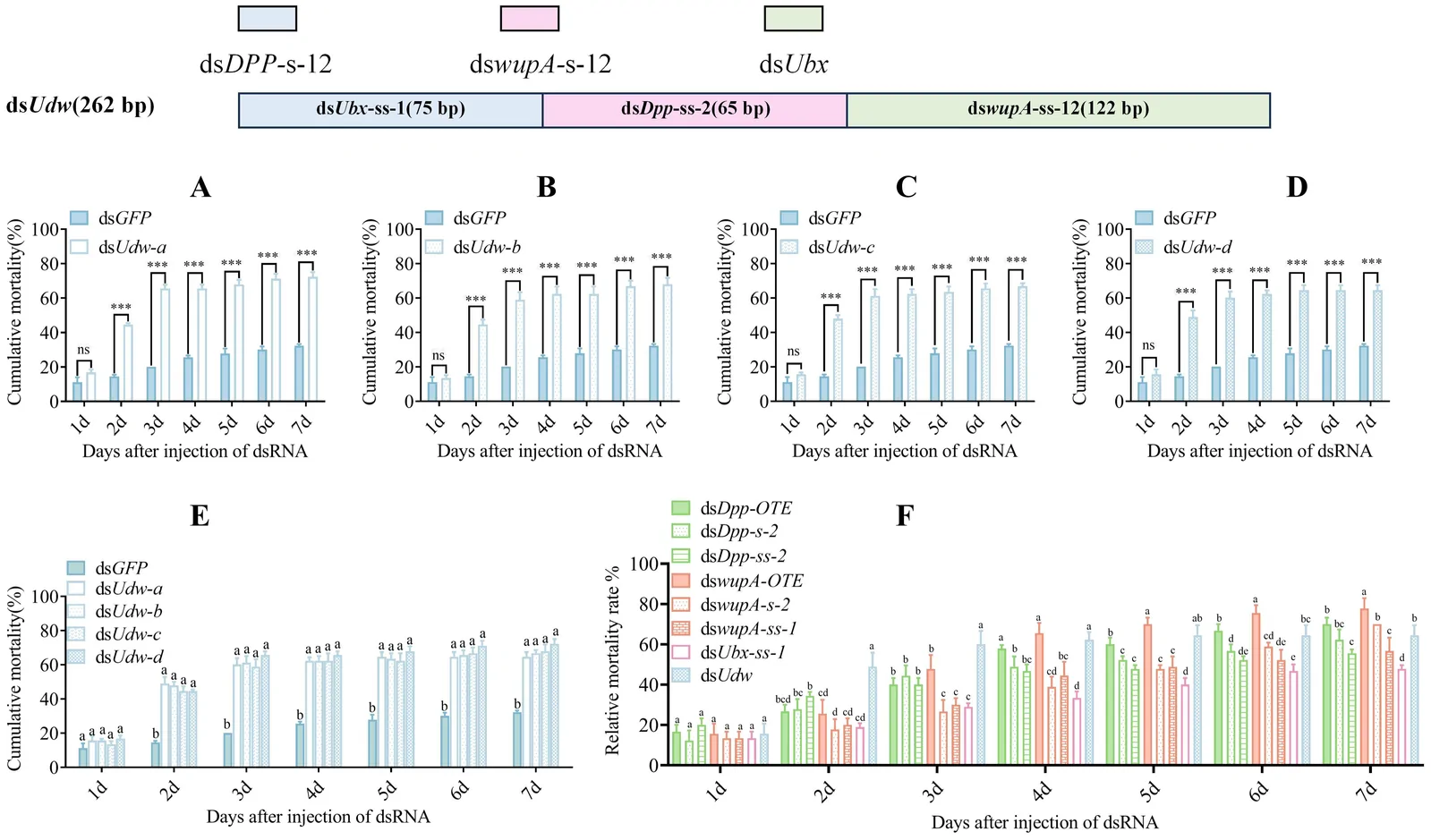
Efficacy verification of the artificially combined sequence *Udw*. Cumulative mortality of 3rd-instar *A. lucorum* nymphs 7 d after microinjection of dsRNA with different concentrations (injection volume: 41.1 nL). **(A)** ds*Udw*-1 (0.5 μg/μL); **(B)** ds*Udw*-2 (1 μg/μL); **(C)** ds*Udw*-3 (3 μg/μL); **(D)** ds*Udw*-4 (5 μg/μL); **(E)** Mortality comparison of 3rd-instar nymphs injected with ds*Udw* at different concentrations; **(F)** Relative mortality comparison between nymphs treated with ds*Udw*-4 and other high-efficiency short dsRNA fragments (*p < 0.05, **p < 0.01, ***p < 0.001, and ns was no significant difference).

As shown in **Figure 5**, dsUdw was diluted to four concentrations: 0.5 μg/μL, 1 μg/μL, 3 μg/μL and 5 μg/μL, named ds*Udw*-a, ds*Udw*-b, ds*Udw*-c and ds*Udw*-d respectively. These constructs were injected into third-instar nymphs of *A. lucorum*, with ds*GFP* serving as the control. Cumulative mortality was recorded daily for seven consecutive days. The 7-day cumulative mortalities of the four treatment groups were 64.44 ± 2.94% (*P* < 0.001), 66.67 ± 1.93% (*P* < 0.001), 67.78 ± 4.01% (*P* < 0.001) and 72.22 ± 2.94% (*P* < 0.001). Mortality increased gradually with rising dsRNA concentration, while no significant difference was observed among treatment groups.

The fragment with the highest lethal effect (ds*Udw*-d, 5 μg/μL) was further compared with other dsRNAs at the same concentration, including off-target optimized fragments (ds*wupA*-OTE, ds*Dpp*-OTE), the most potent s-type fragments (ds*Dpp*-s-2, ds*wupA*-s-2) and the most active ss-type fragments (ds*Dpp*-ss-2, ds*wupA*-ss-1, ds*Ubx*-ss-1). Relative mortality (the difference in mortality between treatment and control groups) was used as the evaluation index. The lethal dynamics comparison revealed that dsUdw-d exhibited significantly higher mortality than all single-gene dsRNAs on the 2nd and 3rd days post injection and reached a peak value of 45.55 ± 2.22% on the 3rd day, indicating a faster insecticidal rate (**Figure 5**).

## 4 Discussion

This study aimed to develop RNA interference (RNAi)-based green control technology for *A. lucorum*, a major agricultural pest. Through target gene screening, off-target risk optimization, and fragment refinement, we successfully constructed an artificially recombinant dsRNA, designated Udw, which exhibits high insecticidal efficacy, enhanced biosafety, and a faster action speed.

Two target genes, *wupA* and *Dpp*, were identified via microinjection, both achieving mortality rates over 90% against *A. lucorum*, consistent with their essential roles in insect growth and development. Fishilevich et al. (41) reported that feeding dsRNA of *wupA* to *Diabrotica virgifera* at 500 ng/cm² resulted in 94.6% mortality, accompanied by destruction of striated muscle fiber structure, impaired locomotion, and inhibited development. In addition, RNAi-mediated silencing of *wupA* may disrupt Malpighian tubule function (42). The *Dpp* gene plays a vital role in wing formation, with functions that vary across species. In *Drosophila melanogaster*, *Dpp* loss-of-function leads to incomplete or missing wing veins (43). Similarly, *Dpp* is highly expressed in both forewings and hindwings of *Athalia rosae*, and its suppression causes complete loss of wing veins (44); In *Precis coenia*, *Dpp* participates in the formation of wing spots and veins. Disruption of *Dpp* function also results in wing malformation in *Nilaparvata lugens* (45). The strong lethal phenotypes indicate that *wupA* and *Dpp* are ideal candidate targets for RNAi. Furthermore, *Ubx* was identified as another effective target, with a mortality rate above 50%; *Ubx* mutations in *Tribolium castaneum* cause abnormal elytron morphology (46); In contrast, silencing *PP1* produced no significant lethal effect in our study, differing from a previous report on *Halyomorpha halys* in which over 70% mortality was achieved with 1 μg dsRNA (47). We suggest that the discrepancy may result from interspecific variation in RNAi efficiency, differences in injection dosage (approximately 0.2 μg in the present study), or the *in vivo* stability of dsRNA (48). These results underscore the necessity of empirical validation when applying RNAi targets across different insect species.

Systematic off-target analysis of target fragments was performed, an essential prerequisite for the field application of RNAi-based biopesticides. Following the criterion that continuous sequence matches of >19 bp may trigger off-target effects (40), we precisely removed risky sites homologous to non-target organisms—particularly vertebrates and beneficial insects—via BLAST alignment. The optimized fragments *wupA*-OTE and *Dpp*-OTE retained potent insecticidal activity while showing improved biosafety. Notably, we defined broad-spectrum target sites that share homology exclusively with other agricultural pests, including aphids, *Thrips palmi*, and bed bugs, and these sequences were intentionally retained. This finding provides valuable structural elements and theoretical support for developing broad-spectrum RNAi products against multiple pest species. However, fragments that aligned with both non-target organisms and pests were also considered off-target and were excluded. Therefore, rigorous scrutiny is required in evaluating broad-spectrum target sites to ensure that selected fragments possess broad-spectrum potential while maintaining relative specificity and safety. In addition, because the cleavage specificity of dsRNA differs among organisms, even if an off-target site or a broad-spectrum target site exists, it may not be functional in practical applications if the corresponding siRNA cannot be generated by Dicer processing. Nevertheless, attention should remain on the potential off-target risks of broad-spectrum target sites to beneficial organisms.

We thoroughly investigated the effects of dsRNA length and target position on RNAi efficiency. Distinct silencing efficiency was observed among dsRNA fragments derived from different regions of the same gene. For instance, ds*Dpp*-s-1 and ds*Dpp*-s-2 exhibited prominent insecticidal activity, whereas ds*Dpp*-s-3 and ds*Dpp*-s-4 showed no obvious effect. Li et al. (13) designed three dsRNA fragments targeting the *V-ATPase E* gene of *N. lugens* and found that fragments covering the 3’-coding and non-coding regions exerted the highest silencing efficiency, whereas those located in the 5’-coding region worked poorly. Tusun et al. (14) constructed three dsRNA fragments targeting different regions of the *JHEH* gene in *A. lucorum*, and all significantly reduced the survival of third-instar nymphs. In general, insecticidal activity declined as dsRNA length decreased from 300–360 bp to 30–90 bp, which is consistent with the conclusion that long dsRNA fragments possess higher efficacy in *Drosophila* cell lines (8). Nevertheless, several short fragments shorter than 100 bp, such as ds*Dpp*-ss-2 (65 bp) and ds*wupA*-ss-1 (66 bp), still maintained high lethal activity. These effective short dsRNAs are favorable for reducing synthesis costs and minimizing potential off-target risks. In addition, some ss-class short fragments shorter than 100 bp retained remarkable lethal activity in this study, while other fragments of the same length showed no obvious RNAi effects. Such differences may be closely related to the local secondary structure of target mRNAs and the cleavage preference of Dicer enzymes (49, 50).

The core innovation of this study lies in the construction of a multi-target recombinant dsRNA Udw by concatenating effective short fragments from different genes. Compared with single-target dsRNAs, Udw has two remarkable advantages. Firstly, it achieves a faster insecticidal speed. The relative mortality of Udw-treated nymphs was significantly higher than all single-gene groups on days 2 and 3 post-injection and reached the peak on day 3. This may be because Udw simultaneously interferes with three independent and critical physiological processes to accelerate mortality: *Ubx* (regulating morphological development), *Dpp* (signal transduction), and *wupA* (muscle contraction). Secondly, Udw shows high dosage sensitivity. Even at a 10-fold diluted concentration (0.5 μg/μL), it still caused over 64% mortality. This allows lower application dosage in field conditions, thereby cutting production costs and alleviating environmental residue. Furthermore, the multi-target strategy can theoretically delay the development of pest resistance caused by mutation of a single target gene (51).

In summary, this study provides a promising candidate fragment Udw for the control of *A. lucorum*. More importantly, we established a complete technical workflow covering target screening, biosafety optimization and functional validation, and proposed a novel design strategy for multi-target recombinant dsRNA. These findings lay a solid theoretical foundation and offer new perspectives for developing efficient, safe, economical and fast-acting RNAi pesticides. However, all current experiments were conducted via microinjection under laboratory conditions. Further research including feeding and spray bioassays, as well as field and greenhouse evaluations on control efficacy and non-target organism safety, is required in follow-up studies.

## Data Availability

The original contributions presented in the study are included in the article/**Supplementary Material**. Further inquiries can be directed to the corresponding author.
