# Supplementary material for: Screening, optimization and artificial recombination of dsRNA fragments for RNAi-mediated pest resistance in Apolygus lucorum
Source: Front Insect Sci. 2026 Aug 18;6:1912427. doi: 10.3389/finsc.2026.1912427 (PMC13529733; doi:10.3389/finsc.2026.1912427)
Supplement: Supplementary file 1 [file Table1.docx]

| **targeted insects** | **Sequence ID** | **target Site 1(bp)** | **target Site 2(bp)** | **E**  **value** | **Identities** |
| --- | --- | --- | --- | --- | --- |
| *Sipha flava*  co-targeted insects（*DPP*） | XM_025569616.1 | 1548-1569 | 1613-1641 | 2E-59 | 84% |
| Cimex lectularius  co-targeted insects（*DPP*） | XM_014392004.2 | 888-918 | 941-960 | 2E-48 | 78% |
| *Diuraphis* *noxia*  co-targeted insects（*DPP*） | XM_015512753.1 | 644-672 |  | 6E-49 | 81% |
| *Rhopalosiphum* *maidis*  co-targeted insects（*DPP*） | XM_026949515.1 | 1504-1522 |  | 2E-45 | 80% |
| *Melanaphis* *sacchari*  co-targeted insects（*DPP*） | XM_025335613.1 | 1511-1537 |  | 8E-41 | 79% |
| *Ficedula* *albicollis*  off-targeted insects（*DPP*） | XM_005047779.1 | 1111-1129 |  | 1E-35 | 80% |
| *Lonchura* *striata*  off-targeted insects（*DPP*） | XM_021538433.1 | 1222-1240 |  | 7E-34 | 80% |
| *Centropristis* *striata*  off-targeted insects（*DPP*） | XM_059352682.1 | 1243-1261 |  | 4E-50 | 81% |
| *Cololabis* *saira*  off-targeted insects（*DPP*） | XM_061743408.1 | 1470-1488 |  | 4E-50 | 81% |
| *Malurus* *melanocephalus*  off-targeted insects（*DPP*） | XM_057380954.1 | 1484-1502 |  | 5E-49 | 80% |
| *Prinia* *subflava*  off-targeted insects（*DPP*） | XM_063399663.1 | 1232-1250 |  | 5E-49 | 80% |
| *Ficedula* *albicollis*  off-targeted insects（*DPP*） | XM_005047779.1 | 1114-1132 |  | 5E-49 | 80% |
| *Pseudoliparis* *swirei*  off-targeted insects（*DPP*） | XM_056427125.1 | 1443-1461 |  | 2E-48 | 80% |
| *Argentina* *silus*  off-targeted insects（*DPP*） | XM_083684004.1 | 1524-1542 |  | 2E-48 | 80% |
| *Poromitra* *crassiceps*  off-targeted insects（*DPP*） | XM_083632815.1 | 1573-1591 |  | 2E-48 | 80% |
| *Astatotilapia* *burtoni*  off-targeted insects（*DPP*） | LT989955.1 | 1490-1508 |  | 6E-48 | 80% |
| *Micromesistius* *poutassou*  off-targeted insects（*DPP*） | XM_084481801.1 | 1553-1571 |  | 6E-48 | 80% |
| *Agelaius* *tricolor*  off-targeted insects（*DPP*） | XM_071426117.1 | 11231-1249 |  | 2E-47 | 80% |
| *Erithacus* *rubecula*  off-targeted insects（*DPP*） | XM_079309765.1 | 1252-1270 |  | 2E-47 | 80% |
| *Melospiza* *georgiana*  off-targeted insects（*DPP*） | XM_058026515.1 | 1187-1205 |  | 2E-47 | 80% |
| *Ammospiza* *nelsoni*  off-targeted insects（*DPP*） | XM_059475242.1 | 1248-1266 |  | 2E-47 | 80% |
| *Agelaius* *phoeniceus*  off-targeted insects（*DPP*） | XM_054635506.1 | 1045-1063 |  | 2E-47 | 80% |
| *Ammospiza* *caudacuta*  off-targeted insects（*DPP*） | XM_0558807034.1 | 1248-1266 |  | 2E-47 | 80% |
| *Agelaius* *tricolor*  off-targeted insects（*DPP*） | XM_071426119.1 | 1045-1063 |  | 2E-47 | 80% |
| *Zonotrichia* *leucophrys*  off-targeted insects（*DPP*） | XM_064714480.1 | 1236-1254 |  | 2E-47 | 80% |
| *Haemorhous* *mexicanus*  off-targeted insects（*DPP*） | XM_059850632.1 | 1250-1268 |  | 2E-47 | 80% |
| *Lonchura* *striata*  off-targeted insects（*DPP*） | XM_021538433.1 | 1222-1240 |  | 2E-47 | 80% |
| *Fringilla* *coelebs*  off-targeted insects（*DPP*） | XM_084207617.1 | 1255-1273 |  | 2E-47 | 80% |
| *Melospiza* *melodia*  off-targeted insects（*DPP*） | XM_063159647.1 | 1203-1221 |  | 2E-47 | 80% |
| *Taeniopygia* *guttata*  off-targeted insects（*DPP*） | XM_030275258.4 | 1232-1250 |  | 2E-47 | 80% |
| *Thrips* *palmi*  co-targeted insects（*wupA*） | XM_034391211.1 | 557-588 | 626-646 | 5E-60 | 92% |
| *Plutella* *xylostella*  co-targeted insects（*wupA*） | XM_083027466.1 | 510-538 |  | 8E-44 | 84% |
| *Halyomorpha* *halys*  co-targeted insects（*wupA*） | XM_014433491.3 | 445-467 |  | 1E-61 | 79% |
| *Cimex* *lectularius*  co-targeted insects（*wupA*） | XM_014399596.1 | 604-623 |  | 5E-60 | 78% |
| *lps* *typographus*  co-targeted insects（*wupA*） | XM_081989830.1 | 642-659 |  | 2E-58 | 81% |
| *Nilaparvata* *lugens*  co-targeted insects（*wupA*） | XM_039423876.1 | 1098-1116 | 1203-1228 | 1E-55 | 86% |
| *Frankliniella* *occidentalis*  co-targeted insects（*wupA*） | XM_026423759.2 | 648-668 |  | 5E-54 | 77% |
| *Dendroctonus* *ponderosae*  co-targeted insects（*wupA*） | BT128535.1 | 522-547 |  | 6E-53 | 80% |
| *Loxostege* *sticticalis*  off-targeted insects（*wupA*） | EU339105.1 | 554-598 |  | 1E-41 | 84% |
| *Bombyx* *mandarina*  off-targeted insects（*wupA*） | XM_028187055.1 | 590-642 |  | 1E-40 | 85% |
| *Amyelois* *transitella*  off-targeted insects（*wupA*） | XM_013330278.2 | 602-630 |  | 2E-39 | 84% |
| *Manduca* *sexta*  off-targeted insects（*wupA*） | XM_030173370.2 | 629-634 |  | 7E-39 | 79% |
| *Bombyx* *mori*  off-targeted insects（*wupA*） | NM_001043830.1 | 558-589 |  | 1E-40 | 85% |
| *Dinoponera* *quadricep*  co-targeted insects（*ubx*） | XM_014630865.1 | 1267-1295 | 1351-1370 | 4E-51 | 81% |
| *Aedes* *aegypti*  co-targeted insects（*ubx*） | XM_021838503.1 | 1492-1523 | 1588-1610 | 9E-63 | 85% |
| *Aedes* *albopictus*  co-targeted insects（*ubx*） | XM_019678240.3 | 2369-2400 | 2465-2487 | 9E-63 | 85% |
| *Rhagovelia* *plumbea*  co-targeted insects（*ubx*） | MG999805.1 | 639-667 |  | 3E-48 | 81% |
| *Pogonomyrmex* *barbatus*  co-targeted insects（*ubx*） | XM_025218575.1 | 1531-1559 |  | 2E-40 | 79% |
| *Euwallacea* *fornicatus*  co-targeted insects（*ubx*） | XM_066294803.1 | 955-980 |  | 6E-73 | 80% |
| *Bactrocera* *latifrons*  co-targeted insects（*ubx*） | XM_018927209.1 | 2170-2189 |  | 6E-73 | 83% |
| *Armigeres* *subalbatus*  co-targeted insects（*ubx*） | XM_062698285.1 | 1843-1874 |  | 2E-72 | 84% |
| *Euwallacea* *similis*  co-targeted insects（*ubx*） | XM_066392227.1 | 961-986 |  | 7E-72 | 80% |
| *Sitophilus* *oryzae*  co-targeted insects（*ubx*） | XM_030902333.1 | 953-972 | 995-1014 | 3E-70 | 81% |
| *Bactrocera* *dorsalis*  co-targeted insects（*ubx*） | XM_029550125.1 | 2435-2454 |  | 3E-70 | 82% |
| *Cephus* *cinctus*  co-targeted insects（*ubx*） | XM_015745033.2 | 2132-2150 |  | 4E-68 | 78% |
| *Limulus* *polyphemus*  off-targeted insects（*ubx*） | XM_013938649.2 | 789-817 |  | 4E-41 | 80% |
| *Orussus* *abietinus*  off-targeted insects（*ubx*） | XM_012421147.1 | 1681-1709 |  | 2E-55 | 82% |
| *Bombus* *vosnesenskii*  off-targeted insects（*ubx*） | XR_004495211.1 | 2735-2763 |  | 3E-52 | 80% |
| *Harpegnathos* *saltator*  off-targeted insects（*ubx*） | XM_011150519.3 | 1845-1873 |  | 2E-49 | 81% |
| *Cyphomyrmex* *costatus*  off-targeted insects（*ubx*） | XM_018539916.1 | 652-680 |  | 3E-47 | 80% |
| *Vollenhovia* *emeryi*  off-targeted insects（*ubx*） | XM_012015285.1 | 1943-1971 |  | 3E-47 | 80% |
| *Trachymyrmex* *cornetzi*  off-targeted insects（*ubx*） | XM_018515513.1 | 1494-1522 |  | 4E-46 | 80% |
| *Monomorium* *pharaonis*  off-targeted insects（*ubx*） | XM_028190067.2 | 1208-1236 | 1295-1314 | 7E-44 | 80% |
| *Atta* *colombica*  off-targeted insects（*ubx*） | XM_018198049.1 | 963-974 | 1050-1069 | 9E-43 | 80% |
| *Acromyrmex* *echinatior*  off-targeted insects（*ubx*） | XM_011058039.1 | 805-833 | 892-911 | 9E-43 | 80% |
| *Solenopsis* *invicta*  off-targeted insects（*ubx*） | XM_011157106.3 | 1547-1575 |  | 3E-42 | 80% |
| *Osmia* *lignaria*  off-targeted insects（*ubx*） | XM_034326971.2 | 10107-10135 |  | 2E-39 | 78% |
| *Frankliniella* *occidentalis*  off-targeted insects（*ubx*） | XM_026417130.2 | 2357-2380 |  | 2E-39 | 83% |
| *Osmia* *bicornis*  off-targeted insects（*ubx*） | XM_046290186.1 | 10040-10062 |  | 2E-39 | 78% |
| *Pseudomyrmex* *gracilis*  off-targeted insects（ubx） | XM_020424416.1 | 3225-3253 |  | 4E-69 | 80% |
| *Drosophila* *ananassae*  off-targeted insects（*ubx*） | XM_032451764.2 | 1665-1691 |  | 1E-68 | 82% |
| *Drosophila* *subpulchrella*  off-targeted insects（*ubx*） | XM_037867197.1 | 1692-1711 |  | 1E-68 | 82% |
| *Drosophila* *gunungcola*  off-targete*d* insects（*ubx*） | XM_052980750.1 | 1637-1656 | 1775-1794 | 1E-68 | 82% |
| *Drosophila* *suzukii*  off-targeted insects（*ubx*） | XM_036817883.3 | 1725-1744 |  | 1E-68 | 82% |
| *Drosophila* *Yakuba*  off-targeted insects（*ubx*） | XM_039375306.1 | 1701-1720 | 1840-1858 | 1E-68 | 82% |
| *Drosophila* *bipectinate*  off-targeted insects（*ubx*） | XM_017247668.3 | 1654-1673 | 1726-1748 | 1E-68 | 82% |
| *Drosophila* *takahashii*  off-targeted insects（*ubx*） | XM_017149029.3 | 1700-1719 | 1839-1857 | 1E-67 | 81% |
| *Drosophila* *elegans*  off-targeted insects（*ubx*） | XM_017278730.2 | 963-982 | 1102-1120 | 1E-67 | 81% |
| *Drosophila* *ficusphila*  off-targeted insects（*ubx*） | XM_017184144.2 | 1725-1744 | 1860-1879 | 1E-67 | 81% |
| *Diorhabda* *carinulata*  off-targeted insects（*ubx*） | XM_057804128.1 | 1157-1179 |  | 1E-67 | 80% |
| *Drosophila* *biarmpies*  off-targeted insects（*ubx*） | XM_017094229.3 | 1762-1781 | 1900-1919 | 1E-67 | 81% |
| *Drosophila* *teissieri*  off-targeted insects（*ubx*） | XM_043796368.1 | 1702-1721 | 1840-1859 | 1E-67 | 81% |
